# Haemagglutinin antigen selectively targeted to chicken CD83 overcomes interference from maternally derived antibodies in chickens

**DOI:** 10.1038/s41541-022-00448-2

**Published:** 2022-03-03

**Authors:** Angita Shrestha, Rick Meeuws, Jean-Remy Sadeyen, Pengxiang Chang, Marielle Van Hulten, Munir Iqbal

**Affiliations:** 1 The Pirbright Institute, Ash Road, Pirbright, Woking, Surrey, GU24 0NF UK; 2 Department of Zoology, Peter Medawar Building, South Parks Road, University of Oxford, Oxford, OX1 3SY UK; 3 Global Poultry R&D Biologicals Boxmeer, Intervet International BV, MSD Animal Health, Wim De Körverstraat 35, 5831 AN Boxmeer, The Netherlands; 4 Present Address: GlaxoSmithKline, Gunnels Wood Rd, Stevenage, SG1 2NY UK

**Keywords:** Vaccines, Protein vaccines

## Abstract

Maternally derived antibodies (MDAs) are important for protecting chickens against pathogens in the neonatal stage however, they often interfere with vaccine performance. Here, we investigated the effects of MDAs on a targeted antigen delivery vaccine (TADV), which is developed by conjugating H9 subtype avian influenza virus haemagglutinin (HA) antigen to single chain fragment variable (scFv) antibodies specific for the chicken antigen presenting cell receptor CD83. Groups of 1-day-old chickens carrying high levels of MDAs (MDA++) and 14-day old chickens carrying medium levels of MDAs (MDA+) were immunised with TADV (rH9HA-CD83 scFv), untargeted rH9HA or inactivated H9N2 vaccines. Immunogenicity in these vaccinated chickens was compared using haemagglutination inhibition (HI) and enzyme-linked immunosorbent assays (ELISA). The results showed that the TADV (rH9HA-CD83 scFv) induced significantly higher levels of H9HA-specific antibody titres compared to the untargeted rH9HA and inactivated H9N2 vaccines in MDA++ and MDA+ chickens. Overall, the data demonstrates immune responses induced by TADV are not affected by the MDA in chickens.

## Introduction

Avian influenza viruses (AIV) are one of the major threats to poultry production, inflicting severe losses to poultry as well as posing credible zoonotic and pandemic threats^1^. Outbreaks of low pathogenicity avian influenza viruses (LPAIV) and high pathogenicity avian influenza viruses (HPAIV) have resulted in severe economic losses to the poultry industry due to expenses associated with culling and quarantine, emergency vaccination programmes and loss of consumer confidence^2–4^. Despite years of research and control efforts, the number and severity of AIV outbreaks continue to increase around the world^5^. Surveillance and strict biosecurity are the key first line of defence against AIV. However, the biosecurity systems of current poultry farms are being repeatedly compromised and continued incursion of novel strains are causing disease outbreaks^6^. Therefore, vaccination is used as an additional measure to control AIV. Experimental and field studies have shown that vaccines can protect against clinical signs and death, reduce shedding of virus, and prevent contact transmission of the virus^7–9^. Previously, blanket vaccination against AIV was primarily used in enzootic countries like Vietnam, Egypt and Indonesia however, nowadays more targeted and risk-based strategies are used to reduce the cost and increase the efficiency of the vaccination programmes^10^. The enzootic prevalence of H9N2 LPAIV across Asia, Middle East and North Africa has resulted in the routine AIV vaccination programmes using inactivated AIV vaccines in several countries in this region^11–13^. Furthermore, the prevalence of Asian lineage H5Nx HPAIV has compelled many countries to enforce AIV vaccination policies. In the European Union, “preventive” vaccination against H5 viruses has been allowed in outdoor poultry^14^. As a consequence, progeny chickens from vaccinated hens will have maternally derived antibodies (MDAs) in the first few weeks after hatch^15^.

The MDAs are vertically transferred from mothers to the hatching progeny^16^. In chickens, serum IgY antibodies (functional equivalent of mammalian IgG) are transported from the hen’s blood stream into the oocyte (which becomes the egg yolk) through a specific receptor that recognises the Fc domain^17,18^. During the embryo development, the IgY antibodies are transported from the egg yolk into the embryo’s systemic circulation through receptor-mediated transcytosis^19,20^. This transport of IgY antibodies has been documented as early as seven days into incubation, and peaks towards the last couple of days before hatch^21,22^. Such MDAs passed through the egg to the hatching progeny (passive immunity) are important for immunity against pathogens in the neonatal stage, when immunocompetence is not fully developed in the chicks^23–25^. Furthermore, MDAs avoid the energetic cost of fighting off infections by the offspring and allow chicks to retain energy for growth and further development of the immune system^26,27^. However, MDAs can often interfere with the vaccination of young chickens by masking the specific epitopes of the vaccine antigen and thus, reducing the antigen presentation^28^. Additionally, it has been shown that B cell activation can be inhibited through a cross-link between B cell receptors (BCR) and the Fcγ-receptors IIB present on B cells by vaccine-MDA complexes^29^. This affects the induction of active immune responses following vaccination^30^. Several studies have reported poor induction of antibody titres after vaccination of chickens with MDAs^31–33^. Therefore, MDA interference is of major concern for the poultry industry, as it is one of the reasons for the failure of various poultry vaccines.

The majority of commercially available AIV vaccines are inactivated whole influenza vaccines, largely produced in embryonated hen’s eggs. The use of such conventional inactivated vaccines remains a challenge due to the need for repeated administration, difficulty in differentiating infected from the vaccinated animals (DIVA) and interference from MDAs^14^. Various strategies have been developed in the recent years to overcome these challenges and enhance the overall immunogenicity of vaccines. One such strategy is the recombinant targeted antigen delivery vaccine (TADV) whereby protective antigens are selectively delivered to professional antigen presenting cells (APCs) such as dendritic cells (DC), macrophages and B cells^34^. Such antigen targeting can be done by either chemically conjugating the antigen to monoclonal antibody (mAb) specific for selected APC receptors or by genetic engineering in which the antigen is fused to antibody fragments such as single chain fragment variable (scFv) antibodies specific for the APC receptors^35^. Several studies have explored antibody-based antigen targeting to mammalian APC’s via Dec205, CD11c, CD40, Clec9A and MHC II receptors^36–40^. The first in-human study of a protein vaccine targeting APCs was conducted with CDX-1401 vaccine (Celldex Therapeutics Inc., New Haven, Connecticut, USA) which is an anti-cancer vaccine targeting the human Dec205 receptor. This vaccine was proven to be safe and efficacious in phase I of clinical trials^41^. Moreover, the first antigen targeting study in seronegative chickens was also directed towards Dec205 receptor which showed a strong antibody response as early as fourteen days after priming^42^. Recently, we developed a recombinant subunit AIV vaccine by selectively targeting haemagglutinin (HA) antigen of H9N2 AIV to chicken CD83 receptors using single chain fragment variable (scFv) antibodies^43^. The recombinant H9HA Foldon-CD83 scFv vaccine (hereinafter referred to as rH9HA-CD83 scFv) was proven efficacious in specific pathogen free (SPF) birds, conferring faster and higher antibody response, as well as a robust protection against clinical signs, and a higher reduction of virus shedding compared to the untargeted H9HA Foldon vaccine (hereinafter referred to as rH9HA)^43^. The aim of the present work was to investigate the impact of MDAs on the immunogenicity of rH9HA and rH9HA-CD83 scFv vaccines. Here, progeny chickens were generated with H9HA-specific MDAs and vaccinated with rH9HA and rH9HA-CD83 scFv either at day-1 or day-14 post hatch. In addition, the immunogenicity of rH9HA and rH9HA-CD83 scFv was also compared with inactivated whole A/chicken/Pakistan/UDL-01/2008 (UDL 01/08) H9N2 virus vaccine (hereinafter referred to as inactivated H9N2 vaccine) in day-14 old MDA positive chickens.

## Results

### Antibody response in mother hens vaccinated with inactivated H9N2 vaccine

**Fig. 1:**
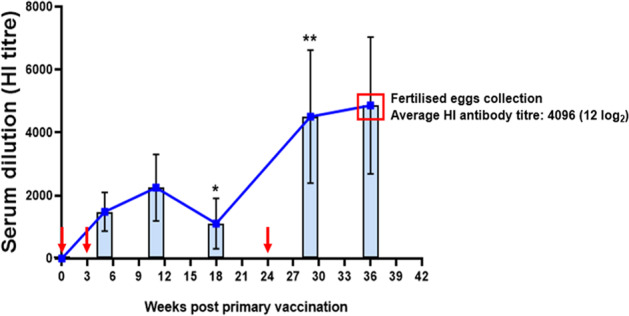
Anti-HA antibody titres measured by HI assay in the serum of mother hens after immunisation with inactivated H9N2 vaccine (UDL 01/08). Hens (White Leghorn) were given prime vaccination at 17 weeks old, and two booster vaccinations at 3 weeks (20 weeks old) and 24 weeks (41 weeks old) post first vaccination (indicated by red arrows). Blood samples were collected at different time points for the serological monitoring of mother hens. The HI titres were expressed as the reciprocal of the highest dilution of serum causing the total inhibition of 4 HA units of UDL 01/08 H9N2 virus hemagglutination activity. Fertilised eggs were collected at 36 weeks (53 weeks old) post first vaccination. The red box indicates average HI antibody titre of mother hens at the time of laying eggs. Data are presented as mean ± SD (*n* = 40) and analysed using paired t-test. The asterisk represents significant difference between the HI antibody titres at 11 and 18 weeks post first vaccine dose, and 18 and 29 weeks post first vaccine dose. **p < 0.01 *p < 0.05.

The SPF White Leghorn Layer hens (*n* = 40) were vaccinated thrice with inactivated H9N2 vaccine. Blood samples were collected at different time points post vaccination for serological monitoring of anti-H9HA antibodies using a standard haemagglutination inhibition (HI) assay. The first blood sampling was carried out at 2 weeks post second vaccine dose (~5 weeks post first dose) where the average HI titre of mother hens was 1351 (10.4 log_2_, Fig. 1). As a significant drop in HI antibody titres was observed in mother hens at 18 weeks post first dose compared to 11 weeks post first dose (*p* < 0.05), a third dose was given at 24 weeks post first dose. This significantly increased the HI antibody titres in mother hens at the next blood sampling timepoint (29 weeks post first dose, *p* < 0.01). Following the third dose of vaccine, the HI antibody titres remained consistent until the last blood sampling timepoint (36 weeks post first dose). Fertilised eggs were collected at 36 weeks post first dose, when the average HI antibody titre of mother hens was 4096 (12 log_2_).

### Anti-H9HA MDA level in the hatchlings reduces significantly by day 14 post hatch

**Fig. 2:**
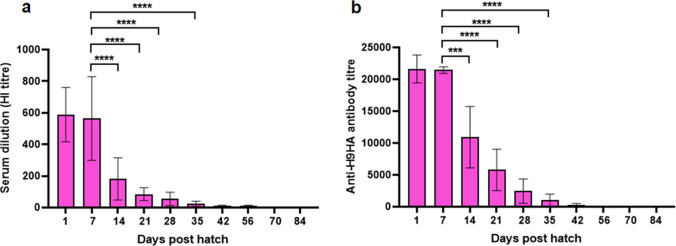
Monitoring the decline of anti-H9HA MDA in the unvaccinated MDA positive control chickens post hatch. **a** Anti-H9HA MDA measured by HI assay at day 1, 7, 14, 21, 28, 35, 42, 56, 70 and 84 post hatch. The HI titres were expressed as the reciprocal of the highest dilution of serum causing the total inhibition of 4 HAU of UDL 01/08 H9N2 virus haemagglutination activity. **b** Anti-H9HA MDA measured by indirect ELISA assay using ID Screen^®^ Influenza H9 Indirect kit (ID. Vet, Cat no: FLUH9S-10P) according to the manufacturer’s protocol. Antibody titres were calculated as 10^(1.3*log^_**10**_^**(Sample/Positive) +3.256)**^. For **a** and **b** data are presented as mean ± SD (*n* = 10) and analysed by one-way ANOVA followed by Tukey’s multiple comparison test. Statistical significance is shown with asterisks. ****p < 0.0001 ***p < 0.001.

Ten hatchlings were sacrificed at 1-day-old and bled to confirm the presence of MDAs. The remaining (*n* = 10) hatchlings per group were bled weekly until 42-days-old and then biweekly until 84-days-old to monitor the decline in the MDA levels. The hatchlings from vaccinated mother hens showed evidence of MDA titres against H9HA in both HI assay and indirect ELISA assay (Fig. 2). At 1-day-old, the hatchlings had an average HI antibody titre of 588 (9.2 log_2_) and anti-H9HA ELISA antibody titre of 21600. The titre dropped slightly (not significant) by day 7 post hatch and was more than halved by day 14 post hatch (average HI titre: 181 (7.5 log_2_), average anti-H9HA ELISA antibody titre:10900). The antibody titres rapidly declined further after that, and by day 35 post hatch the MDA levels were very low (average HI titre: 16 (4 log_2_), average anti-H9HA ELISA antibody titre: ~1000). After day 42 post hatch until the end of experiment at 84 days of age, MDAs were undetectable in both HI and indirect ELISA assays.

### The rH9HA-CD83 scFv vaccine can overcome the interference of high MDA levels (MDA++) compared to rH9HA vaccine

**Fig. 3:**
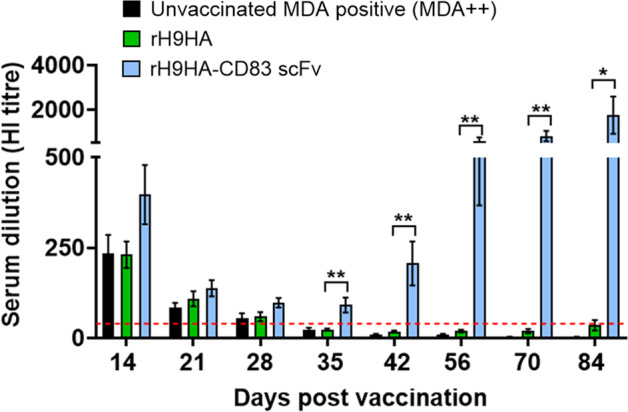
Anti-H9HA antibody titres measured by HI assay in the serum of MDA++ chickens immunised with rH9HA and rH9HA-CD83 scFv vaccines. The HI titres were expressed as the reciprocal of the highest dilution of serum causing the total inhibition of 4 HAU of UDL 01/08 H9N2 virus haemagglutination activity. The red dotted line indicates the predictive protective HI tire of 32. Data are presented as mean ± SEM (*n* = 10 per group) and analysed by one-way ANOVA followed by Tukey’s multiple comparison test. Statistical significance is shown with asterisks. **p < 0.01 *p < 0.05.

The 1-day-old hatchlings had an average HI antibody titre of 588 (9.2 log_2_) and an average anti-H9HA ELISA antibody titre of 21600. Due to these high antibody titres, we designated these chickens as MDA++ (Fig. 2). The chickens in MDA++ groups were immunised at 1-day-old with rH9HA and rH9HA-CD83 scFv vaccines. Blood samples were collected at different time points post vaccination (pv) to follow the antibody responses in chickens with high MDA levels (Fig. 3).

At the first blood sampling point (day 14 pv) the rH9HA-CD83 scFv group showed higher HI antibody titres compared to the rH9HA group, although this was not significant (Fig. 3). The HI antibody titres in rH9HA and unvaccinated MDA positive groups steadily decreased after day 14 pv, and were below the predictive protective value of 32 (5 log_2_)^6^ by day 35 pv. On the contrary, chickens in rH9HA-CD83 scFv group showed an initial decline in HI antibodies until day 28 pv. However, the antibody titres rapidly increased after day 35 pv until the last blood sampling point (day 84 pv). The average HI antibody titre in rH9HA-CD83 scFv group on day 84 pv was 832 (9.7 log_2_) (Fig. 3).

**Fig. 4:**
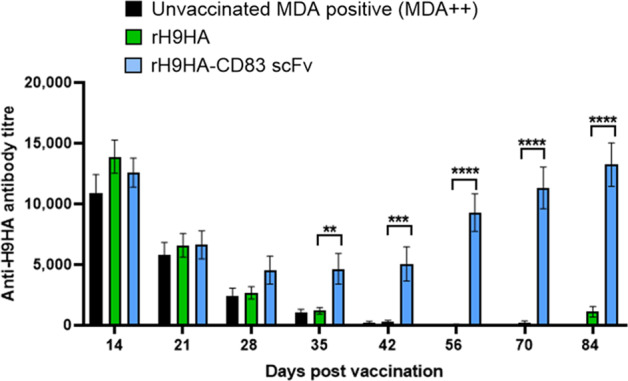
Anti-H9HA antibody titres measured by indirect ELISA assay in the serum of MDA++ chickens immunised with rH9HA and rH9HA-CD83 scFv vaccines. The anti-H9HA antibodies were measured using ID Screen^®^ Influenza H9 Indirect kit (ID. Vet, Cat no: FLUH9S-10P) according to the manufacturer’s protocol. Antibody titres were calculated as 10^(1.3*log^_**10**_^**(Sample/Positive)+3.256**^). Data is presented as mean ± SEM (*n* = 10 per group) and analysed by one-way ANOVA followed by Tukey’s multiple comparison test. Statistical significance is shown with asterisks. ****p < 0.0001 ***p < 0.001 **p < 0.01.

The results obtained from an indirect anti-H9 HA ELISA were very similar to the HI test; there were no significant differences in the anti-H9HA ELISA antibody titres between rH9HA and rH9HA-CD83 scFv groups at day 14 pv (Fig. 4). The antibody titres also showed gradual decline in both the vaccinated groups by day 35 pv. However, at day 35 pv, significantly higher anti-H9HA ELISA antibody titres were recorded in rH9HA-CD83 scFv group compared to the rH9HA group (*p* < 0.01). Furthermore, no anti-H9HA antibodies were detected in most of the chickens in rH9HA group after day 35 pv. Moreover, the chickens in rH9HA-CD83 scFv group presented a rise in the anti-H9HA antibodies after day 35 pv until the last blood sampling point (day 84 pv), mirroring the results observed in the HI assay. This concludes that targeting HA antigen via CD83 scFv significantly increases the vaccine immunogenicity compared to the untargeted HA antigen thus, reducing the susceptibility to MDAs.

### The rH9HA-CD83 scFv vaccine can overcome the interference of medium MDA levels (MDA+) compared to rH9HA and inactivated H9N2 vaccines

The anti-H9HA antibody titres measured by HI and ELISA in the hatchlings showed a significant decline by day 14 post-hatch (average HI titre: 181 (7.5 log_2_), average anti-H9HA ELISA antibody titre:10900, Fig. 2). These chickens were designated as MDA+. The chickens in MDA+ groups were vaccinated at day 14 old with inactivated H9N2 vaccine, rH9HA and rH9HA-CD83 scFv vaccines. Blood samples were collected at different time points after vaccination to follow the antibody responses in chickens with medium MDA levels.

**Fig. 5:**
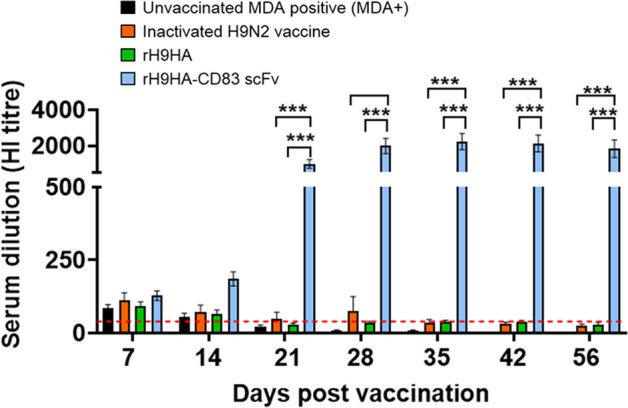
Anti-H9HA antibody titres measured by HI assay in the serum of MDA+ chickens vaccinated with inactivated H9N2 vaccine, rH9HA and rH9HA-CD83 scFv. The HI titres were expressed as the reciprocal of the highest dilution of serum causing the total inhibition of 4 HA units of UDL 01/08 H9N2 virus haemagglutination activity. The red dotted line indicates the predictive protective HI tire of 32. Data are presented as mean ± SEM (*n* = 10 per group) and analysed by one-way ANOVA followed by Tukey’s multiple comparison test. Statistical significance is shown with asterisks. ***p < 0.001.

**Fig. 6:**
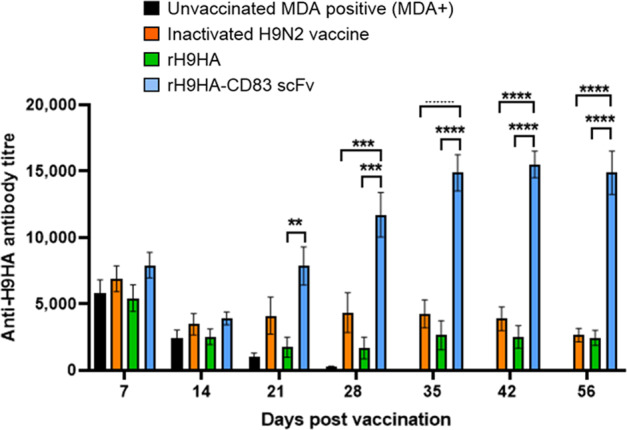
Anti-H9HA antibody titres measured by indirect ELISA assay in the serum of MDA+ chickens vaccinated with inactivated H9N2 vaccine, rH9HA and rH9HA-CD83 scFv vaccines. The anti-H9HA antibodies were measured using ID Screen^®^ Influenza H9 Indirect kit (ID. Vet, Cat no: FLUH9S-10P) according to the manufacturer’s protocol. Antibody titres were calculated as 10^(1.3*log^_**10**_^**(Sample/Positive)+3.256**)^. Data is presented as mean ± SEM (*n*=10 per group) and analysed by one-way ANOVA followed by Tukey’s multiple comparison test. Statistical significance is shown with asterisks. ****p < 0.0001 ***p < 0.001 **p < 0.01.

Unlike the MDA++ group, the chickens in the MDA+ rH9HA-CD83 scFv group showed a rise in the HI antibody titres after day 14 pv, and by the last blood sampling point (day 56 pv), the titres were significantly higher than the inactivated H9N2 vaccine and rH9HA groups (*p* < 0.001, Fig. 5). On the contrary, there was no rise in the HI antibody titres in inactivated H9N2 vaccine and rH9HA groups after day 14 pv. Moreover, the anti-H9HA ELISA antibody titres also showed a similar trend (Fig. 6). The rH9HA-CD83 scFv group demonstrated a strong and steady increase in the anti-H9HA ELISA antibody titres after day 14 pv and the titres were significantly higher than the inactivated H9N2 vaccine and rH9HA groups starting at day 21 pv (*p* < 0.01), day 28 pv (*p* < 0.001), day 35 pv (*p* < 0.0001), day 42 pv (*p* < 0.0001) and day 56 pv (*p* < 0.0001).

Overall, the data suggests that the HI titers and ELISA titers reached similar levels by day 56 pv between MDA+ and MDA++ groups. This suggests that the rH9HA-CD83 scFv vaccine carries almost equal potency in the MDA positive birds vaccinated either at day 1 or day 14 of age. However, more rapid increase in the antibody titres with rH9HA-CD83 scFv in MDA+ group (after day 14 pv) was seen compared to MDA++ group (after day 35 pv) (Figs. 3–6).

## Discussion

Early age chickens with MDAs mount less of an immune response to vaccines compared to those that lack MDAs specific to the antigens constituted in the vaccines. Currently, there is no data available that describe the impact of MDAs on the immunogenicity of recombinant subunit poultry AIV vaccines. Here, we evaluated the ability of recombinant subunit AIV vaccines to overcome MDA interference by using scFv antibodies that target protective antigens to APCs. Using this approach, we developed TADV by conjugating HA antigen of H9N2 virus to scFv antibodies specific for chicken APCs (CD83) receptor. The rH9HA-CD83 scFv vaccine induced a high antibody response and a robust protection against clinical signs after H9N2 virus challenge^43^. To evaluate the ability of rH9HA-CD83 scFv inducing robust immune responses in chickens with MDAs, we produced MDA positive chickens by vaccinating hens White Leghorn with three doses of inactivated H9N2 vaccine (UDL 01/08) adopting commercial vaccination schedule for layer flocks^44^. The progeny chickens from the vaccinated laying hens showed an average HI antibody titre of 588 (9.2 log_2_) at 1-day-old, which was approximately 7-fold lower than the serum HI antibody titre detected in the mothers (average HI:4096 (12 log_2_)), consistent with previous observations^32,44^. The hatchlings displayed a significant drop in the HI antibody titre on day 14 post hatch, and by day 35 post hatch 90% of the chickens showed HI antibody titre below the predicted protective value (HI > 32 (5 log_2_))^6^. A similar trend was observed in the decline of anti-H5HA MDAs in broilers having an average HI titre of 548 (9 log_2_) at 1-day-old^45^. A previous study performed in White Leghorn layers with adjuvanted inactivated H7N3 virus vaccine showed a decrease in MDA-derived HI titres below predicted protective value (HI > 32 (5 log_2_)) by day 14 post hatch^44^. Furthermore, another study using inactivated H5N1 virus vaccine in broiler chickens, displayed a significant decrease in MDA-derived HI titres between day 3 and day 14 post hatch^32,46^. Likewise, hatchlings from Lohmann layer flocks vaccinated with an oil emulsion inactivated H9N2 virus vaccine showed MDAs for 3–4 weeks post hatch^31^. These differences observed in the decline of MDAs in progeny chickens could be attributed to (i) vaccine antigen and formulation (ii) vaccination protocol (iii) the time between immunisation of hens and collection of eggs (iv) number of antibodies present in mother hens during egg laying and (v) chicken breed.

To evaluate the impact of MDAs on the immunogenicity of TADV, the chickens with high H9HA-specific MDA (MDA++, 1-day-old, average HI:588 (9.2 log_2_)) and medium H9HA-specific MDA (MDA+, 14-day-old, average HI:181 (7.5 log_2_)) were vaccinated with rH9HA and rH9HA-CD83 scFv) vaccines. A whole inactivated H9N2 vaccine was also included for comparison in MDA+ group vaccinated at 14-day-old, as 1-day-old vaccination of such vaccine in MDA+ chickens has been previously reported with vaccine failure^47^. In both MDA++ and MDA+ groups, the induction of antibodies after vaccination with rH9HA vaccine was markedly inhibited. Similarly, in MDA+ group the induction of antibodies after vaccination with the inactivated H9N2 vaccine was also inhibited. However, the rH9HA-CD83 scFv vaccine was lesser impacted by both high and medium MDA levels, with HI and anti-H9HA ELISA antibody titres significantly increasing after day 35 pv (MDA++) and day 14 pv (MDA+). Furthermore, a long-lasting antibody response (at least 3 months) was observed when MDA++ chickens were vaccinated with rH9HA-CD83 scFv at 1-day-old. Moreover, a direct comparison between the amount of HA protein in the inactivated H9N2 vaccine and recombinant subunit vaccines could not be made. However, 35 μg of rH9HA and rH9HA-CD83 scFv (equimolar concentration) displayed haemagglutination titre of approximately 256 haemagglutination units (HAU) which is four-fold lower than that present in a single dose of inactivated H9N2 vaccine given to the chickens (1024 HAU).

Previously, live vector vaccines have exhibited variable sensitivity to MDAs^48^. For example, Herpesvirus of Turkey (HVT) expressing AIV HA protein has demonstrated less sensitivity to MDAs and shown to protect chickens even in the presence of MDAs^45,49^. However, other vectors such as Newcastle disease virus (NDV)^46^ and fowl pox virus (FPV) have shown to be more sensitive to MDAs^50^. One of the reasons for HVT overcoming the MDA interference is its ability to replicate in a highly cell-associated manner in lymphocytes which could provide a pathway that can stimulate cell-mediated immunity (CMI)^51,52^. Furthermore, HVT can establish a persistent viremia in chickens for at least 8 weeks following vaccination^53^, continuously expressing the foreign antigen in vivo. This can induce long-lasting immunity even in the presence of MDAs^54^. Moreover, MDAs are shown to exert more inhibition of T helper 2 (Th2)-biased IgG1 than T helper 1 (Th1)-biased IgG2a B cell priming^54^. The inactivated vaccines and recombinant subunit vaccines are known to primarily induce Th2 immunity^55^. Hence, they are thought to be more sensitive to MDA interference. This observation was evident in the present study whereby the immunogenicity of inactivated H9N2 vaccine and recombinant rH9HA vaccine was affected by MDAs. Surprisingly, recombinant rH9HA-CD83 scFv vaccine was able to overcome the MDA interference. The selective targeting of HA antigen to chicken CD83 receptor has shown to stimulate splenocytes in vitro for the production of IFN-γ. Additionally, rH9HA-CD83 scFv has also shown to induce a strong antibody response in vivo. This indicates that rH9HA-CD83 scFv could potentially induce both Th1 and Th2 immune responses^43^. The ability of rH9HA-CD83 scFv to induce Th1 response could have also contributed to its lesser susceptibility to MDA interference. Moreover, it has been demonstrated that vaccines can stimulate B cells for antibody production even in the presence of MDAs if they can stimulate type I interferon (IFN-α)^30^. IFN-α can bind to both interferon receptor and complement receptor 2 (CR2) on B cells. This dual receptor usage leads to a strong positive signal stimulating antibody secretion by B cells even in the presence of MDAs^56^. Thus, vaccines containing adjuvants like LPS and CpG oligonucleotides are able to breakthrough MDAs^30,57^. However, stimulation of chicken splenocytes in vitro by rH9HA-CD83 scFv showed no induction of IFN-α (Supplementary Fig. 1). Another mechanism reported for overcoming the MDAs is through IgM antibodies. It has been reported that antigen-specific IgM antibodies can form a complex with a vaccine antigen and complement protein (C3d), cross-linking the B cell receptor and CR2. Such cross-linking has shown to stimulate B cells in vitro even in the presence of inhibitory IgG^30,58^. Hence, it is possible that vaccines can overcome MDA interference if they can induce higher antigen-specific IgM antibodies. A higher induction of anti-HA IgM antibodies was observed with rH9HA-CD83 scFv vaccine compared to inactivated H9N2 vaccine and rH9HA vaccine in SPF chickens^43^. Therefore, this could be a potential mechanism by which rH9HA-CD83 scFv can overcome MDAs. However, the exact mechanism needs to be further investigated. Overall, the results also indicate that rH9HA-CD83 scFv is highly stable even in the presence of MDAs, unlike rH9HA and inactivated virus vaccines which are easily cleared out. This high stability of rH9HA-CD83 scFv could also explain the rise in antibody titres after the decline in MDAs.

In enzootic countries like Egypt, inactivated AIV vaccines are administered to day 1–5 old broilers^47^. Hatchery vaccination regimes are administered at a preferable 1-day-old to optimise vaccine coverage and achieve early age flock immunity with the use of less resources^59^. However, immunisation of 1-day-old chickens with an inactivated vaccine has faced difficulties in developing good immune responses due to the interference from MDAs, resulting in vaccine failure^47^. The level of MDAs present in chickens at the time of vaccination is a key determinant for the success/failure of the vaccines. Therefore, it is important to determine the optimal age of vaccination according to 1-day-old MDA titres. Studies have reported that vaccination of MDA positive chickens with inactivated AIV vaccine at 7–10 day-old (when MDA titre is low) results in higher immune response and protection compared to 1-day-old vaccination^33^. Several studies have demonstrated the limited value of maternal immunity in protection against AIV infection^32,33^. In some cases, progeny chickens from the vaccinated hens were partially protected from HPAI H5N1 infection only up to 2 weeks after hatch^33^. Hence, it is necessary to vaccinate chickens early, even in the presence of high MDAs to reduce the gap in immunity due to the decline of MDAs and delay in the onset of an active immunity^60^. Therefore, there is a high demand for vaccines that can be administered to 1-day-old chickens to induce an early immune response in high-risk situation. The results from the present study provide evidence that a single dose of the rH9HA-CD83 scFv vaccine can overcome the effects of high MDA levels when administered to 1-day-old chickens. Furthermore, it can also be administered to MDA positive chickens at later age (14-day-old), in situations where 1-day-old vaccination is not applicable.

In conclusion, our results demonstrate that TADV e.g., recombinant rH9HA-CD83 scFv developed by selectively targeting chicken APC receptors induced significantly higher antibody responses in MDA positive chickens compared to the inactivated whole H9N2 AIV vaccine. Therefore, TADV can be regarded as the next generation of poultry vaccine that can overcome the MDA interference. However, further studies are required to test the protective efficacy of TADV against the virus challenge and field performance in the face of MDA in chickens.

## Methods

### Ethics statement

All animal studies and procedures were carried out in strict accordance with Directive 2010/63/EU on the use of animals for scientific research. All animal work was approved by the central committee on animal trials (Centrale Commissie Dierproeven (CCD)) in the Netherlands.

### Viruses, eggs and chickens

A/chicken/Pakistan/UDL 01/2008 H9N2 virus (The Pirbright institute, UK) was used in developing the inactivated H9N2 vaccine.

SPF embryonated chicken eggs (VALO BioMedia) were used for the propagation of UDL 01/08 H9N2 virus.

SPF White Leghorn layer chickens were used for the generation of MDA positive hatchlings. All chickens were housed in isolation rooms with floor pens. All chickens were given food and water ad libitum for the duration of the experiment.

### Vaccine preparation

Two recombinant subunit vaccines; rH9HA and rH9HA-CD83 scFv were produced in *Drosophila melanogaster* Schneider’s (S2) cells and purified using His-tag affinity chromatography^56^. The HA protein used for making both the recombinant subunit vaccines have 98% amino acid sequence similarity to HA of UDL 01/08 H9N2 virus (GenBank accession number: ACP50708.1, HA1: 19–338 and HA2: 339–560). The inactivated H9N2 vaccine was made by propagating UDL 01/08 virus in 10-day-old SPF embryonated chicken eggs. The virus was inactivated chemically using 0.1% β-Propiolactone (BPL, Alfa Aesar) and three blind passages were performed in 10-day old SPF embryonated chicken eggs to confirm inactivation. This was followed by ultracentrifugation at 207,600 × *g* for 2 h at 4 °C for virus concentration. Both the recombinant subunit vaccines and inactivated H9N2 vaccine were formulated as water-in-oil emulsion. The HA titre of inactivated H9N2 vaccine after formulation was 1040 haemagglutination units (HAU)/ml and 5120 HAU/ml for immunisation of mother hens and MDA positive chickens, respectively. All vaccines were stored at 4 °C until needed.

### Vaccination of mother hens and generation of hatchlings with H9HA-specific MDA

**Fig. 7:**
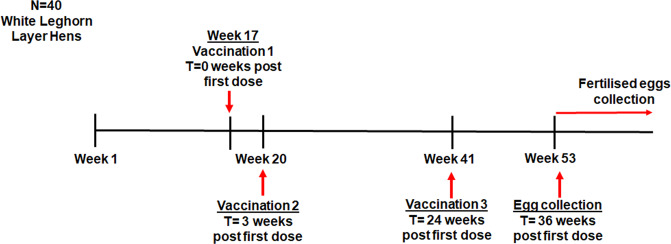
Vaccination schedule of mother hens for the generation of anti-H9HA MDA positive offspring. 40 SPF White Leghorn layer hens were vaccinated via intramuscular route at 17 weeks old with 0.5 ml of inactivated H9N2 vaccine (UDL 01/08, 520 HAU/dose). Two booster vaccinations were given at 20 weeks of age (*T* = 3 weeks post first dose) and 41 weeks of age (*T* = 24 weeks post first dose). Fertilised eggs were collected after 36 weeks post first vaccine dose and set to incubate until hatch.

A group (*n* = 40) of SPF hens (White Leghorn) were immunised with 0.5 ml of inactivated H9N2 vaccine (UDL 01/08) containing 520 HAU/dose. The inactivated H9N2 vaccine was administered by the intramuscular route in the leg muscle. The first dose of vaccine was given to the hens at 17 weeks of age (*T* = 0 weeks post first dose), followed by second and third doses at 20 weeks of age (*T* = 3 weeks post first dose) and 41 weeks of age respectively (*T* = 24 weeks post first dose, Fig. 7). Blood samples were collected from the wing vein of the hens at 5, 11, 18, 29 and 36 weeks after first dose for serological monitoring. Five roosters were included only for egg fertilisation purpose, and therefore were not part of the actual study. Fertilised eggs were collected at 36 weeks after first dose (~11 weeks post third dose) and set to incubate until hatch. At this time point the mother hens (53 weeks of age) contained an average serum HI antibody titre of 4096 (12 log_2_). Ten hatchlings were sacrificed at 1-day-old and blood serum samples were collected to determine the level of MDA in new-born hatchlings at the beginning of the experiment.

### Vaccination of chickens with anti-H9HA MDA and blood sample collection

**Fig. 8:**
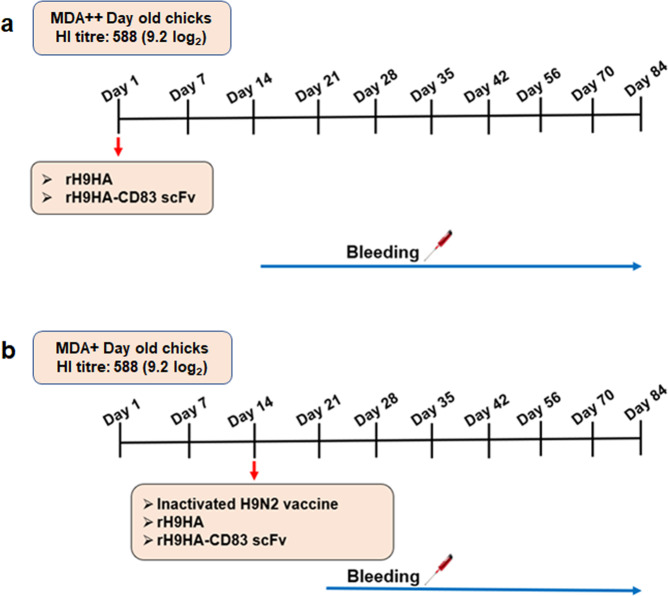
Vaccination schedule of MDA positive chickens. **a** Chickens with an anti-H9HA HI antibody titre of 588 (9.2 log_2_) were designated high MDA positive (MDA++) and vaccinated subcutaneously at 1-day-old with rH9HA (0.2 ml) and rH9HA-CD83 scFv (0.2 ml, 35 μg of rH9HA in equimolar concentration). **b** Chickens with anti-H9HA HI antibody titre of 181 (7.5 log_2_) were designated medium MDA positive (MDA+) and vaccinated subcutaneously at 14-day-old with the inactivated H9N2 vaccine (0.2 ml, 1024 HAU/dose), rH9HA (0.2 ml, 35 μg) and rH9HA-CD83 scFv (0.2 ml, 35 μg of rH9HA in equimolar concentration). Blood samples were collected each week post vaccination.

The MDA positive hatchlings were divided into two groups; high MDA positive (HI titre:588 (9.2 log_2_), MDA++, 1-day-old) and medium MDA positive (HI titre: 181 (7.5 log_2_), MDA+, 14-day-old) (Fig. 8). Groups of 1-day-old MDA++ chickens (*n* = 10 per group) were immunised with 0.2 ml of rH9HA and rH9HA-CD83 scFv vaccines each containing 35 μg of rH9HA protein in equimolar concentration. Furthermore, groups of 14-day-old MDA+ chickens were immunised with 0.2 ml of inactivated H9N2 vaccine (1024 HAU/dose) and 0.2 ml of recombinant rH9HA and rH9HA-CD83 scFv vaccines (concentration same as that given to MDA++ groups). A single vaccination was administered to all the groups via the subcutaneous route. Unvaccinated MDA positive chickens were also included in the study to follow the natural decline in anti-H9HA MDA levels. Blood samples were collected from the wing veins of the chickens at 14, 21, 28, 35, 42, 56, 70- and 84-days post vaccination. For unvaccinated MDA positive control group, blood samples were also collected from newly hatched and 7-day-old chickens by decapitation.

### Haemagglutination inhibition (HI) assay

Whole blood was collected from the vaccinated chickens and the sera separated by centrifugation at 1500 × *g* for 30 min at 4 °C. The HI assay was performed in V-bottomed microtiter plates following World Health Organisation guidelines^61^. Briefly, two-fold serial dilution of the serum was prepared by mixing 25 μl of serum with 25 μl phosphate-buffered saline (PBS). Next, 4 HAU (in 25 μl) of UDL 01/08 H9N2 virus was added to the diluted serum and incubated at 37 °C for 1 h. Finally, 50 μl of 1% chicken red blood cells were added onto the serum-virus mixture and incubated at room temperature for 45 min. The HI titres were expressed as reciprocal of the highest dilution of serum that causes total inhibition of 4 HAU of virus haemagglutination activity.

### Indirect H9HA Enzyme linked immunosorbent assay (ELISA)

The anti-H9HA antibodies in the serum of vaccinated chickens were detected using ID Screen^®^ Influenza H9 Indirect kit (ID. Vet, Cat no: FLUH9S-10P) according to the manufacturer’s protocol. Briefly, 1:500 dilution of the serum samples were prepared in the “dilution buffer", provided by the manufacturer. The plates were then incubated at 21 °C for 1 h. After the incubation step, the plates were washed three times with the wash solution provided, and further incubated with 100 μl of anti-chicken horseradish peroxidase (HRP) conjugate (1:10 dilution, ID. Vet, Cat no: FLUH9S-10P) at 21 °C for 30 min. Plates were again washed with wash solution, then 100 μl of Tetramethylbenzidine (TMB) substrate was added for 15 min. The reaction was stopped using H_2_SO_4_ and read at wavelength 450 nm. Negative and positive controls were provided in the kit and always included in the assay. The test was validated only if the mean value of the positive control optical density (OD) was greater than 0.25, and if the ratio of the mean values of the positive control OD to the negative control OD (OD_PC_/OD_NC_) was greater than 3. For each sample, Sample to Positive ratio (S/P) and antibody titre were calculated using Eqs. 1 and 2 respectively. *S*/*P* > 0.5 and antibody titre >732 were considered positive.

1 $${{S}}/{{P}}\;{{{\mathrm{ratio}}}}:\,\frac{{\it{S}}}{{\it{P}}} = \frac{{{\it{OD}}_{{\it{{\mathrm{Sample}}}}} - {\it{OD}}_{{\it{NC}}}}}{{{\it{OD}}_{{\it{PC}}} - {\it{OD}}_{{\it{NC}}}}}$$

2 $${{{\mathrm{Antibody}}}}\;{{{\mathrm{titre}}}}:{\it{log}}_{10}\left( {{{{\boldsymbol{titre}}}}} \right) = 1.3 \times \log _{10}\left( {\frac{{{{\boldsymbol{S}}}}}{{{{\boldsymbol{P}}}}}} \right) + 3.256$$

$${\it{{\mathrm{Antibody}}}}\;{\it{{\mathrm{titre}}}} = 10^{{\it{log}}_{10}\left( {{{{\mathrm{titre}}}}} \right)}$$

### Statistical analysis

Results are expressed either as mean ± standard deviation (SD) or mean ± standard error of the mean (SEM). Statistical significance (*p*-values) was determined using a one-way ANOVA test followed by post hoc Tukey’s multiple comparison test using Prism 8.3.0 (GraphPad Software). Differences were considered statistically significant if *p* < 0.05.

### Reporting summary

Further information on research design is available in the Nature Research Reporting Summary linked to this article.

## Supplementary information

Supplementary Figure 1

REPORTING SUMMARY

## Data Availability

All data that support the findings of this study are included in the article.
