# Supplementary material for: Haemagglutinin antigen selectively targeted to chicken CD83 overcomes interference from maternally derived antibodies in chickens
Source: NPJ Vaccines. 2022 Mar 3;7:33. doi: 10.1038/s41541-022-00448-2 (PMC8894371; doi:10.1038/s41541-022-00448-2)
Supplement: Supplementary file 1 — Supplementary Figure 1 [file 41541_2022_448_MOESM1_ESM.pdf]

Supplementary Figure

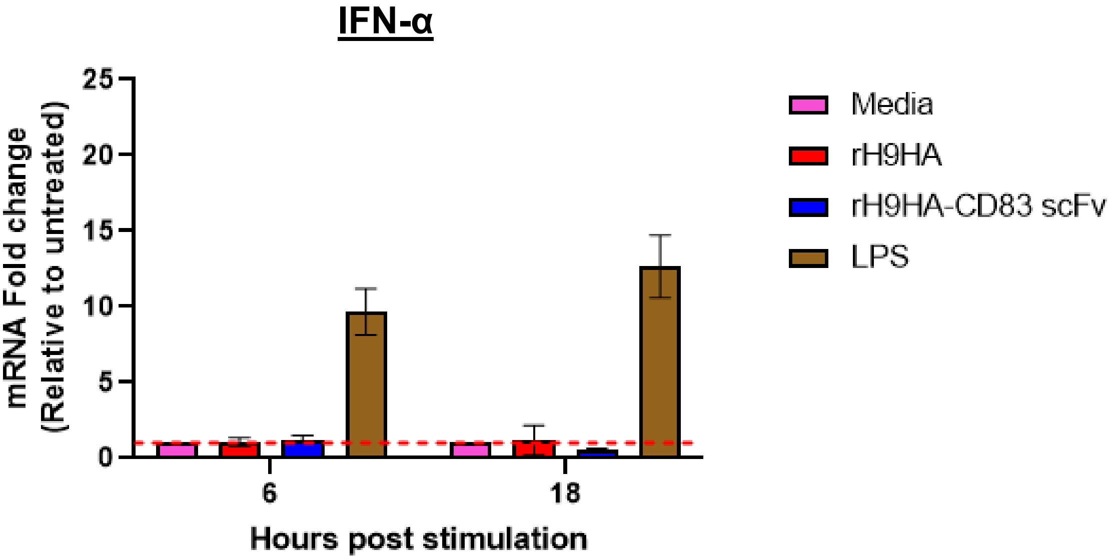

Supplementary Figure 1: Analysis of type I interferon (IFN-α) production by chicken splenocytes upon stimulation with rH9HA and rH9HA-CD83 scFv using quantitative reverse transcription PCR (qRT-PCR). Splenocytes were isolated from the spleen of 3-week-old Specific Pathogen Free chickens using Histopaque 1083 and stimulated with 10 µg of rH9HA and rH9HA-CD83 scFv for 6 hours and 18 hours. Stimulated splenocytes were harvested for RNA extraction and expression level of IFN-α was measured by qRT-PCR. Data were calculated using $2^{-\Delta\Delta CT}$ approach (n-fold change compared to the media only control group) and reported as values normalised to the expression level of a housekeeping gene ribosomal protein lateral stalk subunit PO-1 (RPLPO-1). Data are represented as mean±SD from three independent experiments using different spleen donor chickens and analysed by one-way ANOVA followed by Tukey's multiple comparison test.
