# Supplementary material for: Haemagglutinin antigen selectively targeted to chicken CD83 overcomes interference from maternally derived antibodies in chickens
Source: NPJ Vaccines. 2022 Mar 3;7:33. doi: 10.1038/s41541-022-00448-2 (PMC8894371; doi:10.1038/s41541-022-00448-2)
Supplement: Supplementary file 2 — REPORTING SUMMARY [file 41541_2022_448_MOESM2_ESM.pdf]

nature portfolio

Corresponding author(s): Professor Munir Iqbal

Last updated by author(s): 2021/11/30

Reporting Summary

Nature Portfolio wishes to improve the reproducibility of the work that we publish. This form provides structure for consistency and transparency in reporting. For further information on Nature Portfolio policies, see our Editorial Policies and the Editorial Policy Checklist.

Statistics

For all statistical analyses, confirm that the following items are present in the figure legend, table legend, main text, or Methods section.

n/a	Confirmed	
☐	☒	The exact sample size (n) for each experimental group/condition, given as a discrete number and unit of measurement
☐	☒	A statement on whether measurements were taken from distinct samples or whether the same sample was measured repeatedly
☐	☒	The statistical test(s) used AND whether they are one- or two-sided<br>*Only common tests should be described solely by name; describe more complex techniques in the Methods section.*
☐	☒	A description of all covariates tested
☐	☒	A description of any assumptions or corrections, such as tests of normality and adjustment for multiple comparisons
☐	☒	A full description of the statistical parameters including central tendency (e.g. means) or other basic estimates (e.g. regression coefficient) AND variation (e.g. standard deviation) or associated estimates of uncertainty (e.g. confidence intervals)
☐	☒	For null hypothesis testing, the test statistic (e.g. F, t, r) with confidence intervals, effect sizes, degrees of freedom and P value noted<br>*Give P values as exact values whenever suitable.*
☒	☐	For Bayesian analysis, information on the choice of priors and Markov chain Monte Carlo settings
☒	☐	For hierarchical and complex designs, identification of the appropriate level for tests and full reporting of outcomes
☒	☐	Estimates of effect sizes (e.g. Cohen's d, Pearson's r), indicating how they were calculated

Our web collection on statistics for biologists contains articles on many of the points above.

Software and code

Policy information about availability of computer code

Data collection	No software was used.
Data analysis	No software was used.

For manuscripts utilizing custom algorithms or software that are central to the research but not yet described in published literature, software must be made available to editors and reviewers. We strongly encourage code deposition in a community repository (e.g. GitHub). See the Nature Portfolio guidelines for submitting code & software for further information.

Data

Policy information about availability of data

All manuscripts must include a data availability statement. This statement should provide the following information, where applicable:

- Accession codes, unique identifiers, or web links for publicly available datasets
- A description of any restrictions on data availability
- For clinical datasets or third party data, please ensure that the statement adheres to our policy

All data that support the findings of this study are included in the article.

Field-specific reporting

Please select the one below that is the best fit for your research. If you are not sure, read the appropriate sections before making your selection.

☒ Life sciences ☐ Behavioural & social sciences ☐ Ecological, evolutionary & environmental sciences

For a reference copy of the document with all sections, see nature.com/documents/nr-reporting-summary-flat.pdf

Life sciences study design

All studies must disclose on these points even when the disclosure is negative.

Sample size	The group size and number of replications required are calculated from the minimum number of animals required to achieve statistically robust data. A power of 80% and confidence level of 95% were used to calculate the Sample size.
Data exclusions	No data us excluded for the purpose of analysis.
Replication	All replication of data was successful.
Randomization	Allocation of birds to each study group were random, and observer bias was managed by blinding of treatment groups by numbers only.
Blinding	Only in vitro analysis can confirm differences between the groups therefore, no such observer blinding was required for the experiments.

Reporting for specific materials, systems and methods

We require information from authors about some types of materials, experimental systems and methods used in many studies. Here, indicate whether each material, system or method listed is relevant to your study. If you are not sure if a list item applies to your research, read the appropriate section before selecting a response.

Materials & experimental systems

n/a	Involved in the study	
☒	☐	Antibodies
☐	☒	Eukaryotic cell lines
☒	☐	Palaeontology and archaeology
☐	☒	Animals and other organisms
☒	☐	Human research participants
☒	☐	Clinical data
☒	☐	Dual use research of concern

Methods

n/a	Involved in the study	
☒	☐	ChIP-seq
☒	☐	Flow cytometry
☒	☐	MRI-based neuroimaging

Eukaryotic cell lines

Policy information about cell lines

Cell line source(s)	Drosophila melanogaster S2 cells from Invitrogen Life technologies
Authentication	purchased from commercial source, from Life technologies Thermofisher Scientific.
Mycoplasma contamination	Declere that Cell lienes were not tested for mycoplasma contamination on regular basis.
Commonly misidentified lines (See ICLAC register)	no misidentified lines were used.

Animals and other organisms

Policy information about studies involving animals; ARRIVE guidelines recommended for reporting animal research

Laboratory animals	chicken, White Leghorns mix sex.
Wild animals	Study did not involve wild animals
Field-collected samples	Study did not involve sample collection from the Field.
Ethics oversight	All animal studies and procedures were carried out in strict accordance with Directive 2010/63/EU on the use of animals for scientific research. All animal work was approved by the central committee on animal trials in the Netherlands.

Note that full information on the approval of the study protocol must also be provided in the manuscript.
